# Supplementary material for: Is an Increased Risk of Developing Guillain–Barré Syndrome Associated with Seasonal Influenza Vaccination? A Systematic Review and Meta-Analysis
Source: Vaccines (Basel). 2020 Mar 27;8(2):150. doi: 10.3390/vaccines8020150 (PMC7349742; doi:10.3390/vaccines8020150)
Supplement: Supplementary file 1 [file vaccines-08-00150-s001.pdf]

Supplementary S1

The search was conducted on 29 November 2019. In addition, reference lists of selected articles were also screened to identify other potentially relevant references.

Words for immune illness for a combination with other words in the follow-up:

L1 QUE ?IMMUN? OR ?IMUN? OR ?INFLAMMAT?

Words for autoimmune illness in general:

L2 QUE AUTOIMMUN? OR AUTO(W)IMMUN? OR AUTOIMUN? OR AUTO(W)IMUN? OR AUTOINFLAMMAT? OR AUTO(W)INFLAMMAT?

Words for vaccination:

L3 QUE ?VACCIN? OR IMMUNI!ATION OR ?IMUNI!ATION OR ?IMMUNI!ATION OR ?IMMUNI!ED OR ?IMUNI!ED

Words for cohort studies/clinical trials:

L5 QUE COHORT? OR CASE(W)CONTROL? OR CLINICAL(W)(TRIAL OR STUD?) OR RANDOMI?(4A)(CONTROL? OR CLINIC? OR TRIAL OR STUD?)

Words for the negation of animals:

L6 QUE ANIMAL OR CAT OR DOG OR RABBIT OR GUINEA PIG OR MOUSE OR MICE OR RAT OR CHICKEN OR DUCK OR POULTRY OR GOAT OR SHEEP OR PIG OR PORCINE OR SWINE OR HORSE OR CATTLE OR MONKEY OR RAM OR FISH

Nervous system illnesses:

L17 QUE (GUILLAIN(W)BARRE OR ISAAC## OR DEVIC## OR DEGO###)(2A)(SYNDROM## OR DISEASE).

Table S1. The Newcastle–Ottawa Scale (NOS) score of eligible studies.

Study [Ref.]	Total Score	Selection Score	Comparison Score	Outcome Score
Hurwitz 1981 [11]	6	4	1	1
Kaplan 1982 [12]	6	3	1	2
Lasky 1998 [32]	7	3	2	2
Liu 2003 [33]	5	2	2	1
Hughes 2006 [13]	8	4	2	2
Juurlink 2006 [34]	8	3	2	3
Tam 2007 [4]	8	3	2	3
Stowe 2009 [14]	9	4	2	3
Burwen 2010 [15]	9	4	2	3
Grimaldi-Bensouda 2011 [16]	9	4	2	3
Ho 2012 [35]	8	4	2	2
Tokars 2012 [36]	8	3	2	3
Wise 2012 [17]	7	3	2	2
Greene 2012 [18]	9	4	2	3
Crawford 2012 [19]	8	3	2	3
Baxter 2013 [20]	7	2	2	3
Galeotti 2013 [37]	7	3	2	2
Kwong 2013 [38]	8	4	2	2
McCarthy 2013 [39]	7	3	2	2
Kawai 2014 [21]	8	3	2	3
Chang 2019 [22]	6	2	2	2
Chen 2019 [40]	7	2	2	3
